# Fragment-based screening targeting an open form of the SARS-CoV-2 main protease binding pocket

**DOI:** 10.1107/S2059798324000329

**Published:** 2024-01-30

**Authors:** Chia-Ying Huang, Alexander Metz, Roland Lange, Nadia Artico, Céline Potot, Julien Hazemann, Manon Müller, Marina Dos Santos, Alain Chambovey, Daniel Ritz, Deniz Eris, Solange Meyer, Geoffroy Bourquin, May Sharpe, Aengus Mac Sweeney

**Affiliations:** a Swiss Light Source, Paul Scherrer Institute, 5232 Villigen PSI, Switzerland; b Idorsia Pharmaceuticals Ltd, 4123 Allschwil, Switzerland; F. Hoffmann-La Roche Ltd, Switzerland

**Keywords:** 3CL^pro^, SARS-CoV-2, fragment screening, covalent binders, surface plasmon resonance, X-ray crystallography

## Abstract

To identify starting points for therapeutics targeting SARS-CoV-2, the Paul Scherrer Institute and Idorsia decided to collaboratively perform an X-ray crystallographic fragment screen against its main protease. Fragment-based screening was carried out using crystals with a pronounced open conformation of the substrate-binding pocket. Of 631 soaked fragments, a total of 29 hits bound either in the active site (24 hits), a remote binding pocket (three hits) or at crystal-packing interfaces (two hits). Notably, two fragments with a pose that was sterically incompatible with a more occluded crystal form were identified. Two isatin-based electrophilic fragments bound covalently to the catalytic cysteine residue. The structures also revealed a surprisingly strong influence of the crystal form on the binding pose of three published fragments used as positive controls, with implications for fragment screening by crystallography.

## Abstract 2

X-ray crystallographic screening of SARS-CoV-2 3CL protease resulted in 29 fragment hits, including two isatin-based reversible covalent binders, and revealed a strong influence of the crystal form used for fragment soaking on the bound conformations of three additional reference fragments.

## 1. Introduction

Identifying new chemical leads is a key step in the search to find small-molecule drugs. Fragment-based drug discovery (FBDD) has been developed over the last 20 years and has been increasingly used in drug discovery, especially by the pharmaceutical industry (Knight *et al.*, 2022 ▸). The approach uses a range of different methods to screen fragments (low-molecular-mass ligands obeying the ‘rule of three’; Jhoti *et al.*, 2013 ▸) against a relevant biological target. With the development of automation and higher throughput, X-ray crystallo­graphy has become an efficient method to detect weakly binding fragments and directly identify their binding mode to the target protein. A number of dedicated crystal-based fragment-screening platforms have been developed at synchrotron facilities, including at BESSY II, Diamond Light Source, MAX IV, the European Synchrotron Radiation Facility and the Swiss Light Source (Cipriani *et al.*, 2012 ▸; Douangamath *et al.*, 2021 ▸; Kaminski *et al.*, 2022 ▸; Lima *et al.*, 2020 ▸; Wollenhaupt *et al.*, 2020 ▸).

SARS-CoV-2 has killed millions of people and wreaked havoc on the global economy since its rapid spread in 2019. Despite the development of vaccines and small-molecule drugs, it remains a significant global health burden (Msemburi *et al.*, 2023 ▸). The spread of new variants further increases the medical need for antiviral therapeutics. SARS-CoV-2 encodes several accessory proteins, four structural proteins and 16 nonstructural proteins (NSPs), of which the 3CL protease (main protease, NSP5, 3CL^pro^) is the best studied and is an essential protease for processing SARS-CoV-2 polyproteins (Jin *et al.*, 2020 ▸; Zhang *et al.*, 2020 ▸). The reversible covalent 3CL^pro^ inhibitor PF-07321332 (nirmatrelvir) is used in combination with ritonavir as Paxlovid, an approved treatment for SARS-CoV-2 (Owen *et al.*, 2021 ▸). Additionally, several inhibitors of 3CL^pro^ and other targets are undergoing clinical trials (Mukae *et al.*, 2022 ▸; Lei *et al.*, 2022 ▸). Many of these inhibitors emerged through repurposing, for example of inhibitors of proteases (Pang *et al.*, 2023 ▸). In contrast, fragment screening and FBDD inherently aim at identifying novel starting points.

FBDD studies have identified hundreds of fragments that bind to 3CL^pro^ (Günther *et al.*, 2021 ▸; Douangamath *et al.*, 2020 ▸). The COVID Moonshot project (Boby *et al.*, 2023 ▸; Chan *et al.*, 2021 ▸; Zaidman *et al.*, 2021 ▸) has generated a wealth of small molecules, inhibition data and inhibitor-complex crystal structures. To contribute to the challenging global hit-finding effort, we used a crystal form of 3CL^pro^ with an active-site conformation that is more open and less restricted by crystal packing than that used in most other crystallographic studies of 3CL^pro^ (Günther *et al.*, 2021 ▸; Douangamath *et al.*, 2020 ▸; Supplementary Fig. S1) to screen 631 fragments using the Fast Fragment and Compound Screening (FFCS) facility at the Swiss Light Source (SLS) (Kaminski *et al.*, 2022 ▸, Stegmann *et al.*, 2023 ▸). This open crystal form has been reported previously (Zaidman *et al.*, 2021 ▸; Sutanto *et al.*, 2021 ▸), but was only obtained by co-crystallization with potent, covalent inhibitors and was not previously used for fragment screening. With an overall hit rate of 4.5% (similar to that reported by COVID Moonshot), this crystal-based fragment-screening campaign identified 29 novel fragments in the active site and remote pockets of 3CL^pro^.

Notably, the fragment-screening hits include 3CL^pro^ structures with two fragments based on the reversible, covalent binding motif isatin (indoline-2,3-dione). Isatin is both a known covalent inhibitor of cysteine proteases (Badavath *et al.*, 2022 ▸; Cheke *et al.*, 2022 ▸; Jiang & Hansen, 2011 ▸; Webber *et al.*, 1996 ▸; Zhou *et al.*, 2006 ▸) and an endogenous compound that is present at concentrations of 0.1–10 µ*M* in human tissue (Medvedev *et al.*, 2007 ▸) and the gut microbiome (Medvedev & Buneeva, 2022 ▸). 20 isatin derivatives have been reported by the COVID Moonshot consortium (Morris *et al.*, 2021 ▸), with RapidFire assay IC_50_ values as low as 740 n*M* [SMILES code Cc1nc(CN2C(=O)C(=O)c3cc(Br)ccc32)cs1; Moonshot submission No. LOR-NOR-c954e7ad-1; https://covid.postera.ai/]. The structures of seven isatin inhibitors bound to 3CL^pro^ have been reported on the Fragalysis site and have just been released in the PDB (Boby *et al.*, 2023 ▸) at the time of writing. Isatin-based inhibitors have been modelled in the active site of 3CL^pro^ as both covalent inhibitors (Bao *et al.*, 2023 ▸) and noncovalent inhibitors (ElNaggar *et al.*, 2023 ▸; Badavath *et al.*, 2022 ▸; Liu *et al.*, 2020 ▸), and the structures and surface plasmon resonance (SPR) results reported in this paper confirm a reversible, covalent binding mode.

Moreover, despite the overlap with the results from other fragment-screening campaigns, we report several new motifs and interactions. In conclusion, the fragment-screening results reported in this study provide further information about potential chemical starting points for inhibitors of this pharmaceutically important target.

## 2. Materials and methods

### 2.1. Cloning, protein production and purification of SARS-CoV-2 3CL^pro^

DNA encoding a recombinant fusion protein (see the supporting information) composed of N-terminally hexa­histidine-tagged SUMO and 3CL^pro^ (NC_045512.2, NSP5, YP_009742612, Wuhan-Hu-1) was codon-optimized for expression in *Escherichia coli* and synthesized (GenScript) based on the published expression and crystal structure of 3CL^pro^ (Jin *et al.*, 2020 ▸). The synthetic DNA was cloned into pET-29a(+) using the NdeI and BamHI restriction sites (GenScript) and transformed into *E. coli* BL21(DE3) cells. The protein was expressed overnight (Luria broth medium, 25 µg ml^−1^ kanamycin) at 18°C after induction with 0.5 m*M* isopropyl β-d-1-thiogalactopyranoside (IPTG) at an OD_600_ of approximately 0.7. Overnight cultures were collected by centrifugation and the recovered cell paste was stored at −70°C. 12 g cell paste was resuspended in 20 m*M* Tris–HCl pH 7.8, 150 m*M* NaCl, 5 m*M* imidazole and treated with lysozyme (1 mg ml^−1^; 30 min) and Benzonase (2500 U in 10 m*M* MgCl_2_; 15 min, room temperature). The bacterial cells were lysed by high-pressure homogenization (200 MPa, Microfluidics MP110P, H10Z diamond interaction chamber) and centrifuged for 30 min at 16 000 rev min^−1^ (Fiberlite F21-8 × 50y, maximum r.c.f. 30 392*g*). The hexahistidine SUMO-3CL^pro^ fusion protein was purified by immobilized metal affinity chromatography (IMAC) using a HisTrap column (5 ml, Cytiva) connected to an ÄKTApurifier 100 FPLC system. The histidine-tagged fusion protein was eluted at a flow rate of 2 ml min^−1^ with a linear gradient of increasing imidazole concentration (from 0 to 100% elution buffer over 20 column volumes; the elution buffer consisted of 20 m*M* Tris–HCl pH 7.8, 150 m*M* NaCl, 500 m*M* imidazole). Eluate fractions containing the target protein were combined and concentrated (Amicon, 10 kDa cutoff). The fusion protein was treated with SUMO protease (Sigma–Aldrich; 5 U per milligram of target protein) to liberate 3CL^pro^ with authentic N- and C-termini (Ser1 and Gln306, respectively). The mixture of cleavage products was dialyzed overnight at 4°C using a Slide-A-Lyzer cassette (10 kDa cutoff, Thermo Scientific) in 4 l dialysis buffer (20 m*M* Tris–HCl, 150 m*M* NaCl). The histidine-tagged SUMO protein was separated from nontagged authentic 3CL^pro^ present in the dialysate by IMAC, collecting 3CL^pro^ in the flowthrough. 3CL^pro^ was further purified by size-exclusion chromatography (HiLoad 26/600 Superdex 200) with storage buffer (20 m*M* Tris–HCl, 150 m*M* NaCl, 1 m*M* TCEP, 1 m*M* EDTA). The elution volume of 3CL^pro^ indicated a dimer as the oligomeric state. 3CL^pro^ (97% purity by LC-MS analysis) was concentrated (Amicon, 10 kDa cutoff) to a final protein concentration of 26 mg ml^−1^ (770 µ*M*) and stored at −70°C.

A 3CL^pro^ variant carrying a C-terminal Avi tag (G_307_SGLNDIFEAQK_318_IEWHE) was produced in the same way as the recombinant wild-type protein. To prevent autocleavage of the Avi tag, Gln306 of 3CL^pro^ was replaced by a glutamate (Q306E variant). Western blot analysis with a streptavidin–HRP conjugate confirmed the biotinylation (K_318_) of 3CL^pro^ Q306E during expression in the *E. coli* host strain mediated by the bacterial cell’s endogenous BirA ligase. Biotinylated 3CL^pro^ was used for tethering to SPR sensor chips for small-molecule interaction analysis.

### 2.2. FRET-based 3CL^pro^ proteolytic activity assay

The enzymatic activity of the recombinant SARS-CoV-2 main protease 3CL^pro^ was determined by a fluorescence resonance energy transfer (FRET) assay using a custom-synthesized peptide substrate with (7-methoxycoumarin-4-yl)acetyl (MCA) as the fluorophore and 2,4-dinitrophenyl (DNP) as the fluorescence quencher: MCA-Ala-Val-Leu-Gln-Ser-Gly-Phe-Arg-Lys(DNP)-Lsy-NH_2_, trifluoroacetate salt (Bachem AG, Bubendorf, Switzerland). This peptide-substrate amino-acid sequence corresponds to the nsp4/nsp5 (3CL^pro^) cleavage site. A substrate stock solution (10 m*M*) was prepared in 100% DMSO. 40 µl of a 4 µ*M* substrate solution prepared in H_2_O/0.01% Tween-20) was added to a solution (40 µl) containing 3CL^pro^ to start the enzymatic reaction. The final concentrations of the assay-reaction ingredients (80 µl) were 5 n*M* 3CL^pro^, 2 µ*M* peptide substrate (*K*_m_ = 3.17 µ*M*), 1 m*M* DTT, 1.2% DMSO, 0.01% Tween-20, 25 m*M* Tris–HCl pH 7.4, 0.5 m*M* EDTA. 3CL^pro^ was diluted (to 10 n*M*) from aliquots stored as a stock solution (512 µ*M* in storage buffer at −80°C) in 3CL^pro^ assay buffer (50 m*M* Tris–HCl pH 7.4, 1 m*M* EDTA, 2 m*M* DTT, 0.01% Tween-20). The rate of 3CL^pro^ enzymatic activity (*v*) was determined by monitoring the increase in the fluorescence intensity of reactions at room temperature in black microplates (Nunc 384-well F-bottom) with an Infinite M-100 plate reader (Tecan) using 325 and 400 nm as the wavelengths for excitation and emission, respectively. Test compounds were dissolved in DMSO and initially screened at a final concentration of 25 µ*M*. Threefold serial dilutions (125 µ*M*–6.35 n*M*) of small-molecule test compounds were added to determine the inhibitory potency. IC_50_ values were determined using an in-house evaluation tool (*IC_50_ Studio* with four-parametric fitting and the Hill equation).

### 2.3. Crystallization of SARS-CoV-2 3CL^pro^

Aliquots of purified 3CL^pro^ at 26 mg ml^−1^ in storage buffer were thawed on ice and incubated for 16–18 h at 20°C with a tenfold molar excess of the inhibitor GC376 (Fu *et al.*, 2020 ▸; Ma *et al.*, 2020 ▸). Vapour-diffusion crystallization trials were performed at 20°C using the Morpheus crystallization screen (Molecular Dimensions) with sitting drops consisting of 300 nl each of protein and precipitant solution (Intelli-Plate 96-2, Art Robbins). A single crystal was grown using 30 m*M* sodium nitrate, 30 m*M* disodium hydrogen phosphate, 30 m*M* ammonium sulfate, 100 m*M* MES–imidazole pH 6.5, 20%(*w*/*v*) PEG 550 MME, 10%(*w*/*v*) PEG 20K (Morpheus condition C1) as the precipitant. The crystal was then crushed in the sample well and transferred into a Seed Bead tube (Hampton Research) to obtain a homogeneous suspension of seeds. These seeds were used to crystallize 3CL^pro^ in the absence of the inhibitor GC376 to generate the same crystal form (‘Type 1’; space group *C*2 with unit-cell parameters matching those of PDB entry 7c6u; Fu *et al.*, 2020 ▸). This second round of seeding was considered to be essential to prevent contamination of the final crystals with the potent inhibitor GC376. Interestingly, although GC376-free Type 1 seeds could be generated, their use in the absence of an inhibitor resulted in a different crystal form (‘Type 2’; space group *P*2_1_2_1_2_1_ with unit-cell parameters matching those of PDB entry 7lcr; Vuong *et al.*, 2021 ▸). As Type 1 crystals could not be grown easily without an inhibitor, the Type 2 crystal form was evaluated and selected for subsequent crystallization experiments.

Notably, the pockets in PDB entry 7c6u (Type 1), PDB entry 7lcr (Type 2) and our new fragment-bound Type 2 structures are similarly open, with occasional pocket-widening distortions of the Cys44–Asn51 stretch, and also have a comparably good accessibility with regard to crystal packing (Supplementary Fig. S2).

For reproducible, large-scale crystallization using crystal seeds, 3CL^pro^ was diluted to 8 mg ml^−1^ in 20 m*M* Tris–HCl pH 7.8, 150 m*M* NaCl, 1 m*M* TCEP, 1 m*M* EDTA, 3 m*M* DTT. DTT was added because soaking a small test set of fragments in the absence of a reducing agent revealed oxidation of the active-site cysteine during crystallization and soaking. This solution and the seed-stock solution produced previously were then used to prepare the large-scale, 631-well crystallization. The crystallization trials were set up by transferring 600 nl protein solution and 50 nl seed stock onto an SWISSCI 3-lens crystallization plate and adding 550 nl of the original crystallization condition described above using a Mosquito robotic dispenser (SPT Labtech). The plates were sealed with ClearVue sealing sheets (Molecular Dimensions), incubated and imaged at 20°C with a Rock Imager 1000 (Formulatrix). Hexagonal plate-like crystals appeared after one day and grew to a maximum size of 150 × 80 × 20 µm after three days. These crystals were used for fragment soaking within seven days.

### 2.4. Fast Fragment and Compound Screening (FFCS)

The FFCS pipeline established at the Swiss Light Source was used to perform fragment soaking and screening (Kaminski *et al.*, 2022 ▸; Stegmann *et al.*, 2023 ▸). To determine the optimal DMSO concentration for fragment soaking, the 3CL^pro^ crystals were first soaked for 3 h with 10%, 20% and 30% DMSO using an Echo550 acoustic liquid-handling robot (Labcyte) and were subsequently harvested and measured by X-ray diffraction. A soaking concentration of 20% DMSO was selected based on the results of X-ray diffraction, which showed no deterioration of the data quality.

For crystal-based fragment screening, 631 fragments at a concentration of 100 m*M* in DMSO were prepared in either Echo Qualified 384 low-dead-volume COC microplates (Beckman Coulter) or Echo Qualified 384-well polypropylene microplates (Beckman Coulter), and were then acoustically dispensed into SWISSCI 3-lens crystallization plates at a final fragment concentration of 20 m*M* (20% final concentration of DMSO) using an Echo550 system (Labcyte). The 20% final concentration of DMSO was chosen based on test experiments, which indicated this to be the highest possible DMSO concentration that was tolerated by the 3CL^pro^ crystals. The plates were then sealed and incubated at 20°C. The fragment-soaked crystals were harvested after 3 h, and a Crystal Shifter robot (Oxford Lab Technologies) was used to facilitate and record the harvesting process. No visible damage to the crystals was observed. The crystals were harvested using MiTeGen cryoloops and snap-cooled in liquid nitrogen without further addition of cryoprotectant. The loop-mounted samples were placed in UniPucks for X-ray data collection.

### 2.5. Data collection, processing and structure determination

X-ray diffraction experiments were carried out on the X06SA-PXI protein crystallography beamline at the Swiss Light Source (SLS), Villigen, Switzerland. Data were collected at 100 K using a cryocooled loop in a cryostream. Measurements were made using the Smart Digital User (SDU; Smith *et al.*, 2023 ▸) developed at the SLS with crystal-rotation steps of 0.2° at a speed of 0.01 s per step using an EIGER 16M detector (Dectris) operated in continuous/shutterless data-collection mode. The beam transmission, flux and beam size were 40%, ∼4.4 × 10^11^ photons s^−1^ and 60 × 40 µm, respectively. The estimated X-ray Max dose was 1.65 MGy for a 360° data set (Holton, 2009 ▸).

The data were processed and scaled using *autoPROC* (Vonrhein *et al.*, 2011 ▸) and *XSCALE* (Kabsch, 2010 ▸), respectively. *STARANISO* was used to calculate the diffraction limit and for anisotropic correction. Automated molecular replacement was carried out with *DIMPLE* from *CCP*4 (Agirre *et al.*, 2023 ▸) using the 3CL^pro^ structure (PDB entry 5r83) without any ligand as a template, and *PanDDA* (Pearce *et al.*, 2017 ▸) was used for the automated detection and analysis of weakly bound fragments. *Coot* (Emsley *et al.*, 2010 ▸) was used for model building. *Phenix.refine* (Liebschner *et al.*, 2019 ▸), *BUSTER* (Bricogne *et al.*, 2017 ▸) and *REFMAC* (Murshudov *et al.*, 2011 ▸) were used for refinement of the structures. Data-collection and refinement statistics are reported in Supplementary Table S1. A mosaicity of approximately 0.2° was observed for all data sets.

Figures showing molecular structures were generated with *PyMOL* (version 1.8; Schrödinger). Ligand restraints were generated using *Pyrogen* (Agirre *et al.*, 2023 ▸) or *Grade*2 (Smart *et al.*, 2011 ▸) using the covalently bound chemical structures for cpd-27 to cpd-29.

Similar measurements were carried out at a wavelength of 2.066 Å (6 keV) to locate DMSO molecules using the anomalous signal of sulfur. The beam transmission, flux and beam size were 30%, ∼6.6 × 10^10^ photons s^−1^ and 60 × 40 µm, respectively. The estimated dose was 1.06 MGy for a 360° data set (Holton, 2009 ▸).

All diffraction data and refined models have been deposited in the Protein Data Bank (PDB) with PDB codes 7gre, 7grf, 7grg, 7grh, 7gri, 7grj, 7grk, 7grl, 7grm, 7grn, 7gro, 7grp, 7grq, 7grr, 7grs, 7grt, 7gru, 7grv, 7grw, 7grx, 7gry, 7grz, 7gs0, 7gs1, 7gs2, 7gs3, 7gs4, 7gs5 and 7gs6 as listed in Supplementary Table S1. The SMILES codes of the 29 fragments are reported in Supplementary Table S2.

### 2.6. Surface plasmon resonance (SPR)

SPR experiments were performed using a Biacore T200 equipped with a Series S Sensor Chip SA. Biotinylated 3CL^pro^ Q306E was immobilized on streptavidin covalently attached to a carboxymethyl dextran matrix. The initial conditioning of the surfaces of flow cells 1 and 2 was performed by three 1 min pulses of 1 *M* NaCl, 50 m*M* NaOH solution. The ligand at a concentration of 0.27 mg ml^−1^ in immobilization buffer [10 m*M* HEPES–NaOH, 150 m*M* NaCl, 1 m*M* TCEP, 0.05% polyoxyethylene (20) sorbitan monolaurate (P20) pH 7.4] was immobilized at a density of 3000 RU on flow cell 2 at a flow rate of 5 µl min^−1^; flow cell 1 was left blank to serve as a reference surface. The surfaces were stabilized by a 3 h injection of running buffer (10 m*M* HEPES–NaOH, 150 m*M* NaCl, 1 m*M* TCEP, 0.05% P20, 5% DMSO pH 7.4) at a flow rate of 40 µl min^−1^.

To collect binding data, sample at 625 µ*M* in 10 m*M* HEPES–NaOH, 150 m*M* NaCl, 0.05% P20, 5% DMSO pH 7.4 was injected over the two flow cells at a flow rate of 40 µl min^−1^ and a temperature of 25°C. The complex was allowed to associate and dissociate for 50 and 100 s, respectively, for each sample.

A DMSO correction curve was performed before/after every 104 cycles. Data were collected at a rate of 10 Hz and were analysed using the *Biacore T200 Evaluation* software. 6-Chlorochroman-4-carboxylic acid isoquinolin-4-ylamide was used at 1.25 µ*M* as a positive control. The structure of this inhibitor bound to 3CL^pro^ has previously been solved as Fragalysis entry P0012_0A:ALP-POS-CE760D3F-2 (https://fragalysis.diamond.ac.uk/).

## 3. Results and discussion

**Figure 1:**
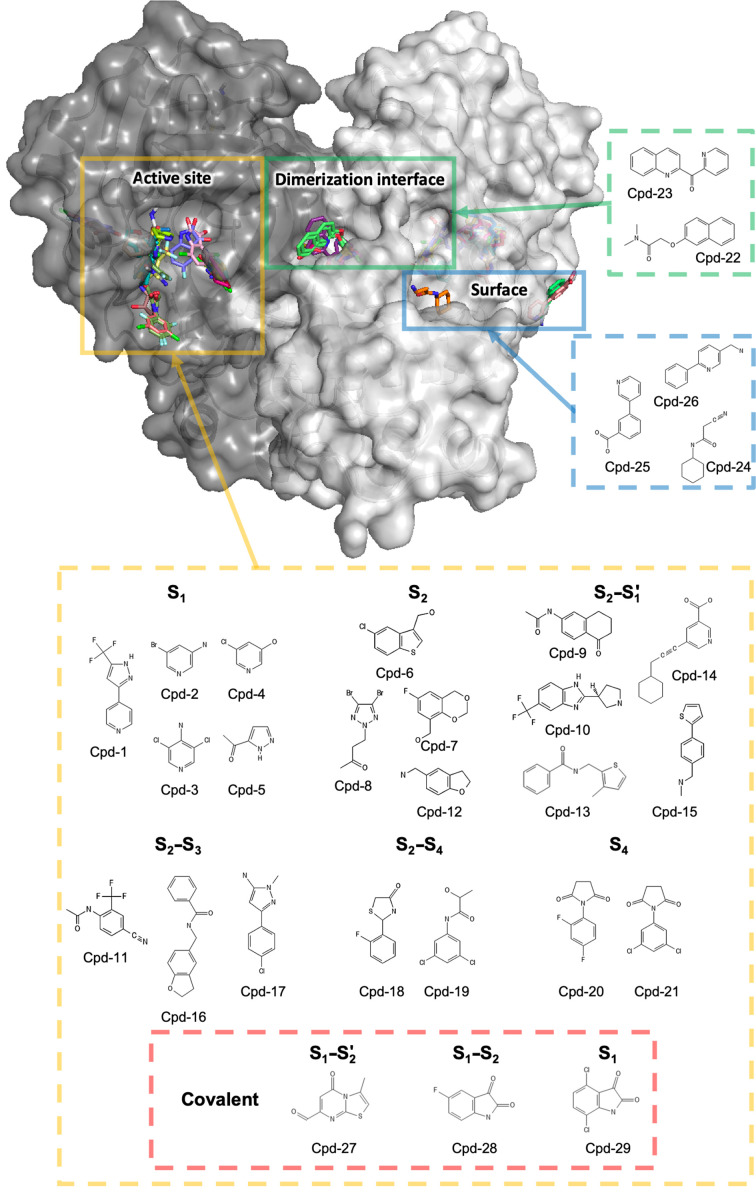
Overall structure of 3CL^pro^ showing the 29 fragment hits. For clarity, only one substrate-binding pocket of the homodimer is shown. The protein is shown in surface representation and fragments are shown in stick representation.

Automated analysis of the fragment-soaked structures using *PanDDA* (Pearce *et al.*, 2017 ▸) was followed by the visual inspection of potential binding events and the full refinement of approximately 60 selected structures. This resulted in a final set of 29 novel fragment-complex structures, including three with borderline electron density for the fragment and three covalently bound compounds. Overviews of the fragment-binding pockets and electron-density maps are shown in Figs. 1 ▸ and 2 ▸, respectively, with the binding site described using the Schechter and Berger notation (Schechter & Berger, 1967 ▸; Supplementary Fig. S3). 24 of the fragments were bound in the substrate-binding pocket, while two fragments were observed in a remote pocket close to the C-terminus (Fig. 1 ▸). The remaining three fragments occupied one of two different remote binding sites partly formed by crystal contacts. Due to their location at crystal-packing interfaces, these compounds (cpd-24 to cpd-26) were considered to be potential artefacts. The fragment structures were refined with a high-resolution cutoff of between 1.47 and 1.92 Å. A limitation of this study is that no quality-control experiments were carried out to detect potential impurities in the commercially available fragments used in order to exclude the risk of incorrectly assigning electron density from an impurity as representing the intended fragment.

### 3.1. Fragment-binding sites and interactions

Representative active-site binders were selected according to their binding mode. The binding of one representative fragment is described for each of the additional binding sites.

#### 3.1.1. Active-site subpockets

The published structure (PDB entry 7mgr; MacDonald *et al.*, 2021 ▸) of the NSP8/9 substrate peptide in complex with the inactive C145A variant of 3CL^pro^ was used to denote the S_1_–S_4_ pockets. The peptide residue Gln5 is bound in S_1_, with Leu4 in S_2_, Lys3 in S_3_ and Val2 in S_4_. Where possible, active-site figures match the standard protease orientation as shown in Supplementary Fig. S3. In this orientation, the N-terminal residues of a peptide substrate are shown on the left and the C-terminal residues on the right.

#### 3.1.2. Open and flexible active site

The crystal form used for these soaking experiments has a more open conformation than, for example, the widely used crystal form (which also belongs to space group *C*2 but with a different crystal packing and unit-cell parameters to Type 1; PDB entry 5r83; Douangamath *et al.*, 2020 ▸; Supplementary Fig. S1). The C_α_ atoms of chain *A* residues 43–52 are shifted by 1.0–1.8 Å from the corresponding residues in PDB entry 5r83, with the Met49 and Ser46 side chains shifted by 3.0 and 2.8 Å, respectively. 3CL^pro^ is present as a homodimer in the asymmetric unit in our structures and as a monomer in the asymmetric unit in the *C*2 form, with the crystallographic twofold axis relating the monomers of the homodimer. As the flexible 3CL^pro^ active site samples many conformations in solution, we believe that the use of different, complementary crystal forms for fragment screening maximizes the diversity of chemical starting points. Furthermore, the flexibility of the open binding pocket is less restricted by crystal contacts.

### 3.2. Noncovalent active-site binders

#### 3.2.1. S_1_ pocket

**Figure 3:**
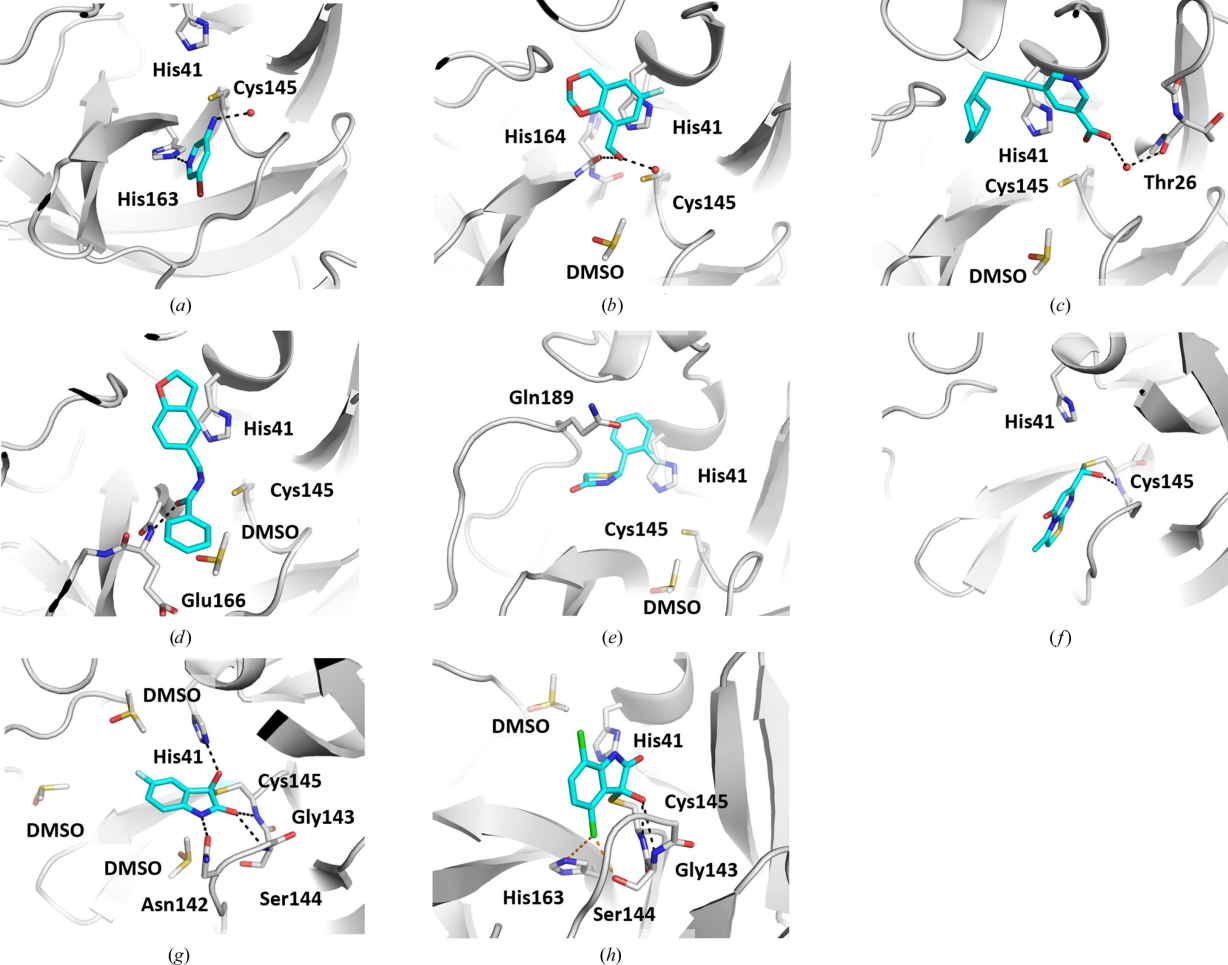
The active-site-bound fragments (*a*) cpd-2, (*b*) cpd-7, (*c*) cpd-14, (*d*) cpd-16, (*e*) cpd-18, (*f*) cpd-27, (*g*) cpd-28 and (*h*) cpd-29. Hydrogen bonds are shown as black dashed lines. Halogen bonds are shown as orange dashed lines in (*h*). The colour code for the stick representations of the fragments is the same as described in Fig. 2 ▸, with C atoms in cyan.

Cpd-2 is bound in the S_1_ pocket (Fig. 3 ▸ *a*). This very small fragment is bound with incomplete occupancy in molecules *A* and *B* (Supplementary Table S2) and its C—Br bond appears to undergo partial radiolysis. In structures with different fragments, we found alternative occupation of the S_1_ pocket by DMSO and the overlapping fragment. In this case, long-wavelength X-ray data collection at 6 keV was also carried out to check the occupancy of DMSO at this location. The absence of a sulfur anomalous peak provided a further strong indication of the cpd-2-bound 3CL^pro^ complex.

Cpd-2 forms a hydrogen bond to His163 and an inter­action with Cys145 S and the adjacent water. A substructure search of the PDB using cpd-2 did not identify any ligands. A search with the Br atom excluded identified a known fragment hit (PDB entry 5re4) with a similar binding pose and hydrogen bond to His163 but with the plane of the ring rotated by approximately 30°. For the remaining compounds, sub­structure searches and similarity searches using the PDB query tool revealed no similar 3CL^pro^ ligands. Other fragments that bind to the S_1_ pocket are cpd-1, cpd-3, cpd-4 and cpd-5. Moreover, cpd-3 and cpd-5 additionally interact with Asn142 O.

#### 3.2.2. S_2_ pocket

Cpd-7 is bound with the dioxane buried in the S_2_ pocket (Fig. 3 ▸ *b*). The fragment forms a single hydrogen bond to His164 O and a face-to-face aryl interaction with His41. The hydroxy group acts as a hydrogen-bond donor, interacting with the His164 backbone carbonyl, but also acts as a hydrogen-bond acceptor in water-mediated hydrogen bonding. Other fragments that bind to the S_2_ pocket are cpd-6, cpd-8 and cpd-12.

#### 3.2.3. S_1_′ pocket

Cpd-14 occupies the S_2_ and 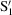 pockets and extends to the entrance to the 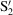 pocket (Fig. 3 ▸ *c*). The cyclohexyl group fits into the hydrophobic S_2_ pocket. The carboxylic acid group forms water-mediated hydrogen-bond interactions with Thr26 and a putative sulfenic acid state of Cys145. The fragment is bound in molecule *A* only. Notably, the 3-carboxy­pyridine group of this fragment is directed into the 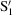 region of the substrate-binding cleft, which is rarely occupied by other fragments. Such a bridging of the catalytic centre by a nonpeptidic motif may be interesting for the design of inhibitors that simultaneously address the prime and nonprime portions of the binding cleft. Positive difference electron density at Cys145 in molecule *A* (only) indicated a modification of the cysteine S atom, possibly an oxidation to form the normally unstable species peroxysulfenic acid, as previously described for 3CL^pro^ (Kneller *et al.*, 2020 ▸).

#### 3.2.4. S_2_ and S_3_ pockets

Cpd-16 extends from S_2_ to S_3_ (Fig. 3 ▸ *d*), forming a face-to-face aryl interaction with His41 and a hydrogen bond between the central amide carbonyl group and the backbone NH of Glu166. The phenyl ring forms no binding interactions with the protein and is more mobile than the rest of the fragment. The fragment is bound in molecule *A* only. Some positive *F*_o_ − *F*_c_ difference electron density adjacent to Cys145 S was not modelled and may be due to partial oxidation.

#### 3.2.5. S_2_ to S_4_ pockets

Cpd-18 extends from S_2_ towards S_4_ (Fig. 3 ▸ *e*). The 2-chlorophenyl group forms a face-to-face aryl interaction with His41. Unlike cpd-16, which occupies the S_2_ and S_3_ pockets, the thiazolidinone carbonyl group of cpd-18 extends directly towards S_4_, displacing the side chain of Gln189. Cpd-6 also occupies the S_4_ pocket and displaces the side chain of Gln189.

#### 3.2.6. S_4_ pocket

**Figure 4:**
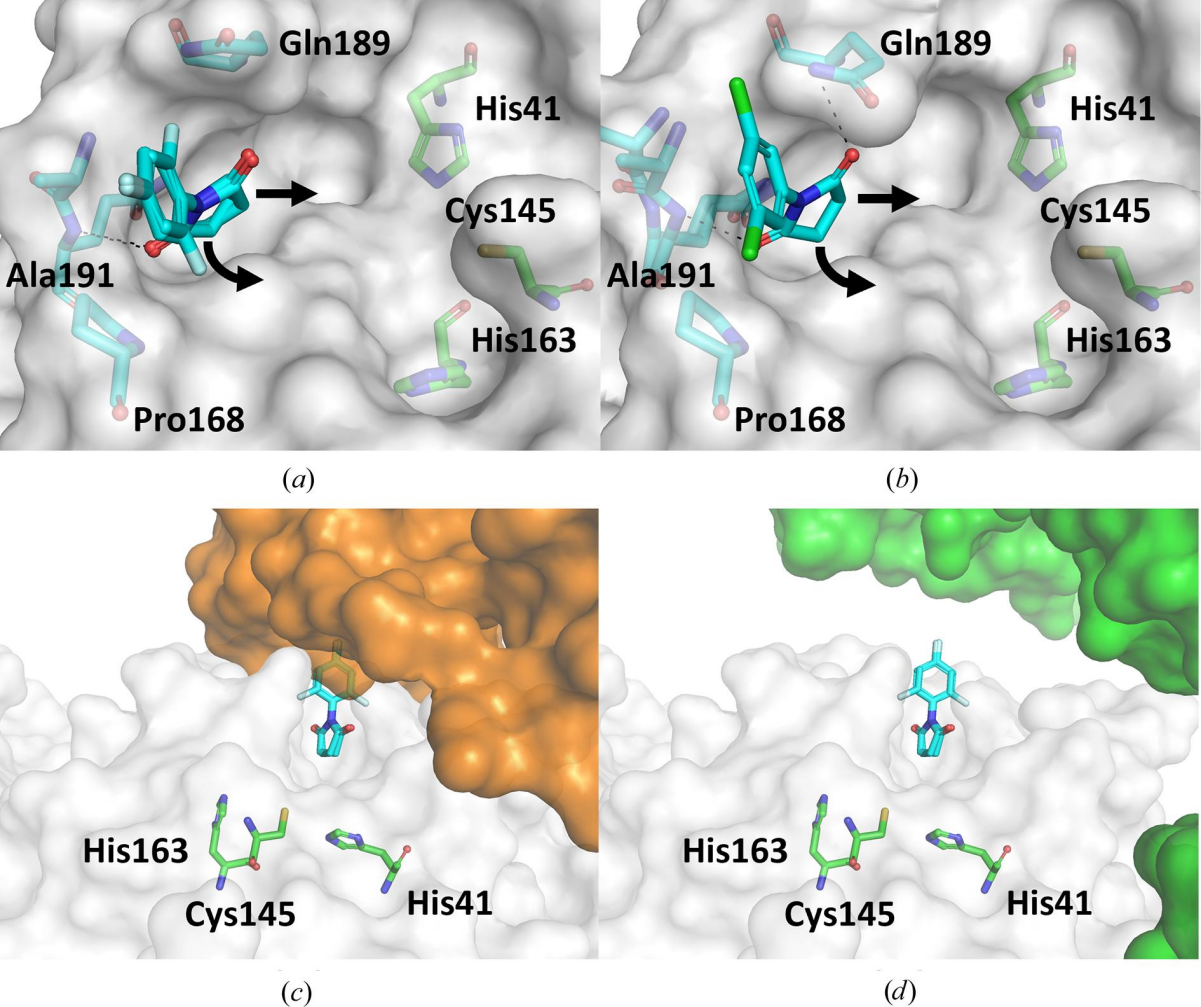
Binding of imide fragments and the blocked binding cleft in the closed crystal form. (*a*) Cpd-20 and (*b*) cpd-21 bind congruently, with their respective N-linked succinimide portions in the S_4_ pocket. Potential exit vectors towards the catalytic centre are indicated by arrows. (*c*) These fragments would clash with a crystal mate (orange surface; PDB entry 5rgr) in the closed crystal form; structures are aligned with respect to the protein monomer binding cpd-20. (*d*) The more open crystal form used in this study allows unobstructed access to the complete binding cleft (the closest crystal mate is shown as a green surface).

Cpd-20 and Cpd-21 bind congruently, with their respective N-linked succinimide portion in the S_4_ pocket (Figs. 4 ▸ *a* and 4 ▸ *b*). This motif has not been observed previously and forms hydrogen-bond interactions with its carbonyl groups to Gln192 NH and the side-chain NH of Gln189. In contrast, the substituted phenyl portions of the fragments form no prominent interactions aside from nonpolar contacts with Pro168 and Ala191. This fact may indicate this site as a strong interaction hotspot for the succinimide motif. Furthermore, these fragments bind without utilizing sub­pockets S_1_–S_3_ closer to the active site, which provide more affinity, yet may possess viable exit vectors towards this region through substitution at the imide N atom or the carbonyl-adjacent carbon position.

The more important observation, however, is that this motif could not have been identified using the closed crystal form used in most other crystallographic screens against 3CL^pro^. In this closed crystal form, the phenyl portion of both fragments would clash with a crystallographic symmetry mate that partially blocks the N-terminal section of the peptide-binding cleft (Fig. 4 ▸ *c*). Thus, it may not be surprising that only very few fragments were reported to be bound to the S_4_ pocket, which then mostly form interactions with the crystal partner, which puts into question the physiological relevance of the observed position. In contrast, the nearest crystal mate of the crystal form used in this study is further away, allowing unobstructed access to the complete binding cleft (Fig. 4 ▸ *d*). Arguably, this crystal mate may also prevent other fragments from accessing the binding cleft, even if their pose does not directly clash with this crystal mate.

### 3.3. Covalent active-site binders

#### 3.3.1. Isatin-based fragments

Cpd-28 and cpd-29 bind covalently to Cys145 via their reactive isatin groups (Figs. 3 ▸ *g* and 3 ▸ *h*, Supplementary Fig. S4). We observed covalent binding of the isatin-based cpd-28 and cpd-29 to the catalytic Cys145 residue in both active sites of the homodimer. Presumably due to the high concentration (20 m*M*) of isatin that was used in the soaking experiment, covalent binding of both compounds to Cys44 was also observed in 3CL^pro^ molecule *B* in the asymmetric unit. Seven structures of isatin-containing fragments bound to 3CL^pro^ have already been solved and shared at https://fragalysis.diamond.ac.uk/, and they have just been released in the PDB (Boby *et al.*, 2023 ▸). Interestingly, a structure of an unrelated COVID-19 protein, the NSP3 macrodomain component of the replication complex, with a noncovalently bound isatin molecule has also been published (PDB entry 5rtf; *PanDDA* event maps have been deposited at https://fragalysis.diamond.ac.uk/; Schuller *et al.*, 2021 ▸).

**Figure 5:**
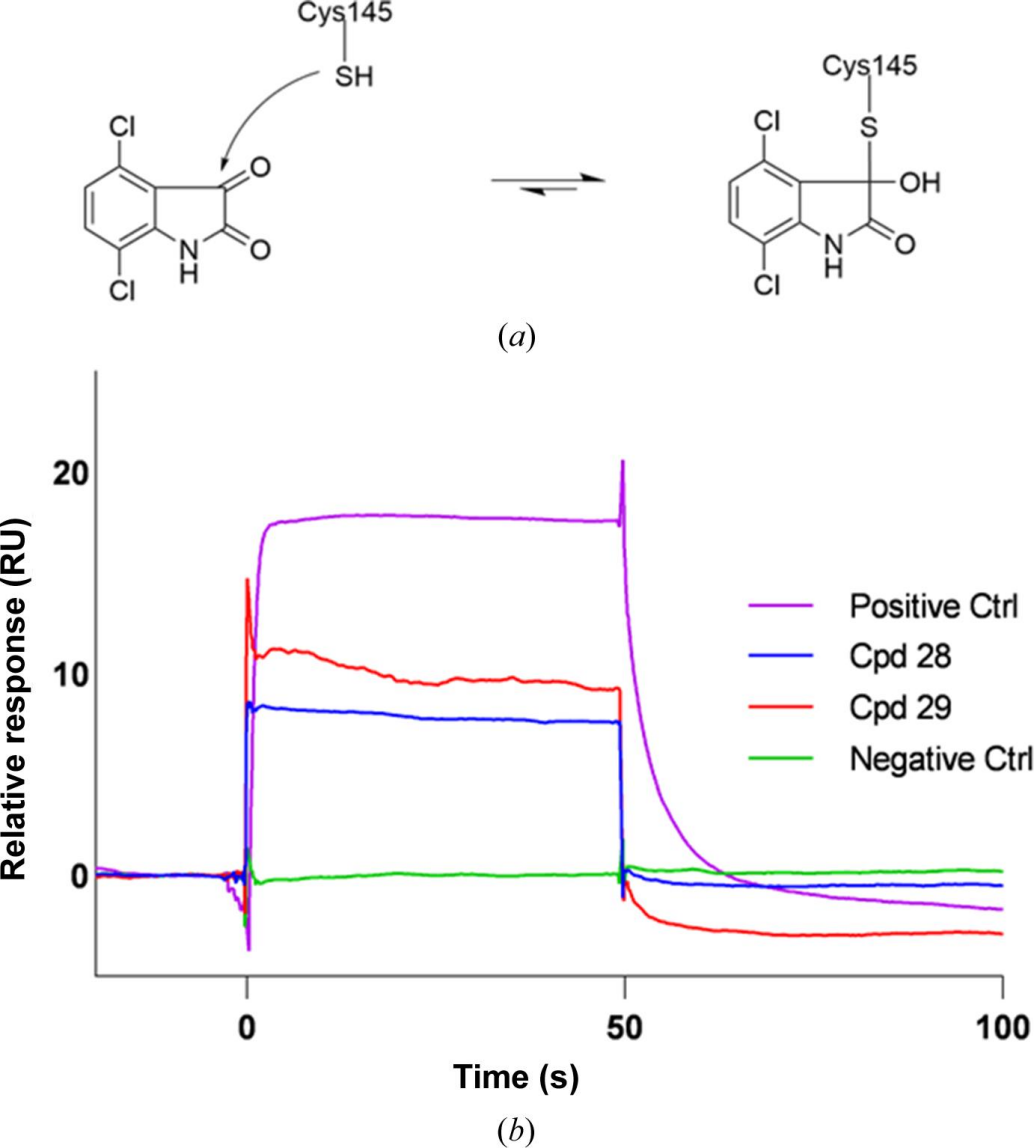
The reversible, covalent inhibition mechanism of 3CL^pro^ by isatin-based inhibitors. (*a*) The reversible covalent-bond formation between an isatin and Cys145. (*b*) A surface plasmon resonance sensorgram showing the rapidly reversible binding of cpd-28 and cpd-29. 6-Chlorochroman-4-carboxylic acid isoquinolin-4-ylamide was used at 1.25 µ*M* as a positive control, and running buffer containing no 3CL^pro^ fragment was used as a negative control.

The reversible binding mode of both inhibitors was confirmed by a FRET-based biochemical assay (Table 1 ▸) and by SPR (Fig. 5 ▸). Similar half-maximal inhibitory concentration (IC_50_) values were observed after pre-incubation of the enzyme and inhibitor for 30 and 60 min, further supporting a reversible binding mode (as the IC_50_ of irreversible inhibitors decreases with an increasing pre-incubation time).

The isatin derivatives cpd-28 and cpd-29 adopt different binding poses. Cpd-29 binds in the S_1_ pocket and forms hydrogen-bond interactions with the backbone N atom of Gly143 and Cys145, while cpd-28 extends towards S_2_ and forms hydrogen bonds to the backbone of Gly143 and the side chain of Asn142. The bulky Cl atom on C atom 4 of cpd-29 (atoms CL1 and C6, respectively) prevents it from adopting the same binding pose as cpd-28 due to a steric clash with the backbone carbonyl group of His164. One of the two Cl atoms of cpd-29 forms halogen bonds to Ser144 and His163. The covalent binding of isatin to Cys145 creates an *R*-configured stereocentre in both bound inhibitors (Fig. 5 ▸). The hydroxy group of the covalently bound cpd-28 forms a hydrogen bond to the His41 side chain. The amino group of isatin makes a hydrogen-bond interaction with the side-chain carbonyl group of Asn142 (Fig. 3 ▸ *g*).

Structures of isatin covalently bound to monoamine oxidase (PDB entry 1oja; Binda *et al.*, 2003 ▸), the cysteine proteases caspase-3 (PDB entry 1gfw; Lee *et al.*, 2000 ▸) and rhinovirus 3C protease (Webber *et al.*, 1996 ▸), and several other proteins have also been published. Isatin derivatives have been reported as both covalent and noncovalent inhibitors of SARS (SARS-1) 3CL^pro^ (Zhou *et al.*, 2006 ▸). Surprisingly, isatin-based inhibitors have been modelled in noncovalent binding poses in the SARS-CoV-2 3CL^pro^ active site and used as the basis for both the *in silico* selection of compounds for screening (Badavath *et al.*, 2022 ▸; Liu *et al.*, 2020 ▸) and modelling-based inhibitor optimization (ElNaggar *et al.*, 2023 ▸). The resulting isatin-based inhibitors have been reported as potent, noncovalent inhibitors (Jiang & Hansen, 2011 ▸). The recently deposited structures provided by the COVID Moonshot, together with the isatin complex structures and SPR results described here, confirm the reversible covalent binding mode of isatin inhibitors to 3CL^pro^ (Table 1 ▸). We hope that these structures, as the first SARS-CoV-2 3CL^pro^ structures deposited in the PDB, will provide clear structural evidence of covalent binding and will serve as templates for future modelling of covalently bound isatins.

In addition to binding at the active site, cpd-28 and cpd-29 also bind to Cys44 of molecule *B*. Unlike Cys145, which is activated by its environment, Cys44 does not take part in the catalytic mechanism, and cpd-28 appears to exist as a mixture of noncovalently bound and covalently bound forms in the Cys44 pocket. The nonselective binding of drugs is clearly undesirable and the design of a more selective isatin-based 3CL^pro^ inhibitor, for example by tuning the electrophilicity of isatin, could be a difficult challenge. Cys44 is close to the substrate-binding site, and the binding of the isatin inhibitors causes a rearrangement of Cys44–Arg60 and Val186–Gly195 and an increase in the size of the S_2_ and S_4_ pockets. In 3CL^pro^ molecule *B*, this rearrangement would result in steric clashes with the neighbouring 3CL^pro^ molecule, and we speculate that this steric hindrance prevents the binding of isatin to Cys44 of 3CL^pro^ molecule *A* in this crystal form.

While isatins and other covalent inhibitors require careful optimization to selectively inhibit the target of interest, inhibitors that bind covalently to the catalytic Cys145 of 3CL^pro^ may be less prone to the development of resistance. As this is a concern for small-molecule antiviral drugs (DeGrace *et al.*, 2022 ▸), substrate-binding pocket analysis was used to highlight the evolutionarily vulnerable regions of 3CL^pro^ that are most likely to tolerate mutations that lead to the development of resistance (Shaqra *et al.*, 2022 ▸). As the catalytic residues of the protease are arguably the most resistant to mutation, we believe that targeting binding through strong interactions with His41 and/or Cys145 of 3CL^pro^ is a promising strategy for the structure-guided optimization of robust inhibitors.

#### 3.3.2. Aldehyde-based fragment

Cpd-27 binds in the S_1_ pocket and partially in the 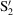 pocket, while forming a reversible covalent bond to the catalytic Cys145 via its aldehyde group (Fig. 3 ▸ *f*, Supplementary Fig. S4). The resulting hemiacetal hydroxy group accepts a hydrogen bond from Cys145 NH and acts as a hydrogen-bond donor, forming a water-mediated interaction with Thr26 (not shown in Fig. 3 ▸ *f*). Aldehydes are known covalent inhibitors of cysteine proteases, and peptidomimetic aldehyde inhibitors were among the first to be described for 3CL^pro^ (Dai *et al.*, 2020 ▸).

### 3.4. Non-active-site binders

#### 3.4.1. Cpd-22 and Cpd-23

**Figure 6:**
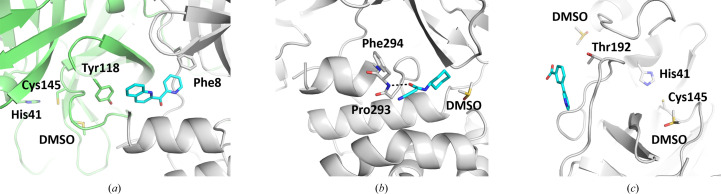
Non-active-site binding fragments (*a*) cpd-23, (*b*) cpd-24 and (*c*) cpd-25.

Cpd-22 and Cpd-23 bind in a pocket that is distinct from the binding site of pelitinib (Fig. 6 ▸ *a*), which is known as the C-terminal dimerization domain (Günther *et al.*, 2021 ▸). This cryptic binding pocket is instead formed by a repositioning of the residues from Ser301 to the C-terminus. Other ligands observed in this pocket include the fragments 1-methyl-*N*-{[(2*S*)-oxolan-2-yl]methyl}-1H-pyrazole-3-carboxamide (PDB entry 5rfa) and 1-(4-fluoro-2-methylphenyl)methanesulfonamide (PDB entry 5rgq; Douangamath *et al.*, 2020 ▸), as well as PEG (PDB entry 8drz) and DMSO (many structures, including PDB entry 7qt6).

#### 3.4.2. Cpd-24, Cpd-25 and Cpd-26

Cpd-24 binds in the same pocket as the compound AT7519 in PDB entry 7aga (Fig. 6 ▸ *b*), which is referred to as the second allosteric pocket (Günther *et al.*, 2021 ▸). Cpd-25 and Cpd-26 bind at the surface of 3CL^pro^ in a pocket that is mainly formed by crystal contacts (Fig. 6 ▸ *c*). Other ligands observed at this location include the fragment 4-amino-*N*-(pyridin-2-yl)benzenesulfonamide (PDB entry 5rf8; Douangamath *et al.*, 2020 ▸) and the buffer components PEG (PDB entry 7kvl) and ethylene glycol (PDB entry 7nf5).

### 3.5. Comparison of five reference compounds in two different crystal forms

**Figure 7:**
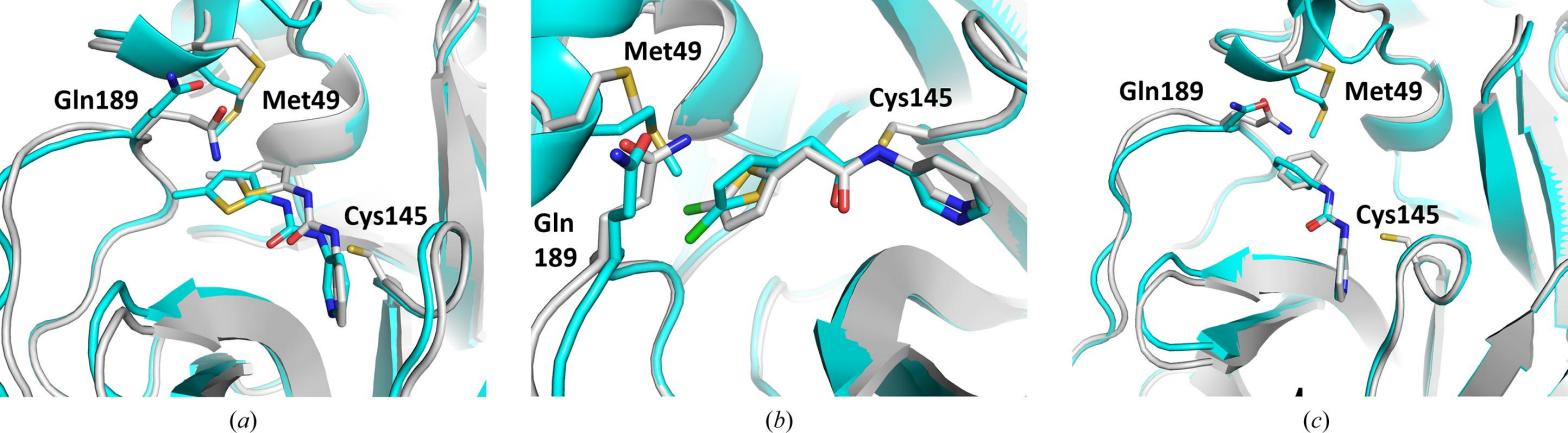
Binding of three fragments in more closed published 3CL^pro^ structures (cyan) compared with this study with a more open 3CL^pro^ conformation (grey). Comparison of the same small molecule in the two different structures shows (*a*) a translation or (*b*, *c*) a rotation of the upper-left ring of fragments corresponding to PDB entries 5rh0, 5rh1 and 5r83, respectively. The position of Met49 shows the more open binding-site conformation used in this study.

The fragments from PDB entries 5rh0, 5rh1, 5rh2, 5r83 and 5rgu were used as positive controls during optimization of the crystal soaking conditions. To check for any influence of the more open active-site conformation used in this study, the fragment-complex structures were compared with the published PDB entries in a different space group (Douangamath *et al.*, 2020 ▸). Notably, three of the five fragments exhibited significant differences in their binding poses (Fig. 7 ▸). While the pyridine moiety of each fragment was fixed via a hydrogen bond to His163, the aromatic ring at the opposite end of each fragment molecule was shifted and rotated by up to 80° (Fig. 7 ▸).

## 4. Conclusion

The significant differences between different structures of the same inhibitor show both the advantages and the potential risks of soaking fragments into crystals for screening or for the structural analysis of validated hits. For flexible binding pockets, restriction of the conformational freedom in the crystal environment may reduce the entropic penalty of binding for a subset of fragments and increase their binding affinity for the crystallized protein. While these hits may bind with a greatly reduced affinity to the free (uncrystallized) target protein, they often provide the first starting points for modelling and potentially for chemical exploration. Equally, the reduced flexibility of the binding pocket may prevent it from adopting the conformation required for the binding of other genuine fragment hits. This may partly explain the low correlation between the fragment hits that are observed when screening by crystallography or other methods (Schiebel *et al.*, 2016 ▸). Finally, although crystal-packing-related artefacts can be minimized by co-crystallization, these results highlight the importance of exploring binding-site flexibility and ligand mobility during structure-guided drug discovery. Room-temperature data collection has been used to probe different active-site conformations and to avoid cryogenic cooling artefacts (Huang *et al.*, 2022 ▸).

In this context, one of the strengths of crystallographic fragment screening is its ability to generate a large number of hits, although this also presents a challenge in medicinal chemistry due to the typically low affinity of these hits. This often leads to hesitancy in their further development, particularly when contrasted with leads from high-throughput screening (HTS) and DNA-encoded library technology (DELT). To effectively utilize the abundance of fragment hits, modern fragment libraries are crafted for rapid and efficient exploration, including compounds that are readily available or can be synthesized quickly (Wollenhaupt *et al.*, 2020 ▸; Cox *et al.*, 2016 ▸). Additionally, computational methods in structure-guided drug design are essential in order to efficiently select and prioritize promising fragment analogs (Boby *et al.*, 2023 ▸; Metz *et al.*, 2021 ▸), enabling focused development with minimal effort. Furthermore, crystallographic fragment screening is invaluable for broader applications, such as assessing target druggability and identifying interaction motifs (Wood *et al.*, 2019 ▸).

In summary, we report a crystallographic fragment screen against 3CL^pro^ using a crystal form that is less obstructed by crystal packing and has a more open substrate-binding cleft conformation than previously used crystal forms. We identified a number of new or varied motifs and binding interactions with the potential to be instrumental in structure-guided drug discovery. In particular, we identified fragments that could not have been found with more closed crystal forms due to steric overlap, but we also revealed varied binding poses of known crystallographic binders in the flexible binding cleft. These observations demonstrate the implications of the chosen crystal form for fragment screening and subsequent structure-guided design. The subsequent use of co-crystallization, different crystal forms and/or room-temperature data collection, together with an awareness of the potential influence of the crystal form, may maximize the value of fragment structures in drug-discovery projects.

## Supplementary Material

PDB reference: SARS-CoV-2 main protease, complex with cpd-1, 7gre

PDB reference: complex with cpd-2, 7grf

PDB reference: complex with cpd-3, 7grg

PDB reference: complex with cpd-4, 7grh

PDB reference: complex with cpd-5, 7gri

PDB reference: complex with cpd-6, 7grj

PDB reference: complex with cpd-7, 7grk

PDB reference: complex with cpd-8, 7grl

PDB reference: complex with cpd-9, 7grm

PDB reference: complex with cpd-10, 7grn

PDB reference: complex with cpd-11, 7gro

PDB reference: complex with cpd-12, 7grp

PDB reference: complex with cpd-13, 7grq

PDB reference: complex with cpd-14, 7grr

PDB reference: complex with cpd-15, 7grs

PDB reference: complex with cpd-16, 7grt

PDB reference: complex with cpd-17, 7gru

PDB reference: complex with cpd-18, 7grv

PDB reference: complex with cpd-19, 7grw

PDB reference: complex with cpd-20, 7grx

PDB reference: complex with cpd-21, 7gry

PDB reference: complex with cpd-22, 7grz

PDB reference: complex with cpd-23, 7gs0

PDB reference: complex with cpd-24, 7gs1

PDB reference: complex with cpd-25, 7gs2

PDB reference: complex with cpd-26, 7gs3

PDB reference: complex with cpd-27, 7gs4

PDB reference: complex with cpd-28, 7gs5

PDB reference: complex with cpd-29, 7gs6

Supplmentary information and Supplementary Figures and Tables. DOI: 10.1107/S2059798324000329/ud5050sup1.pdf

## Figures and Tables

**Figure 2:**
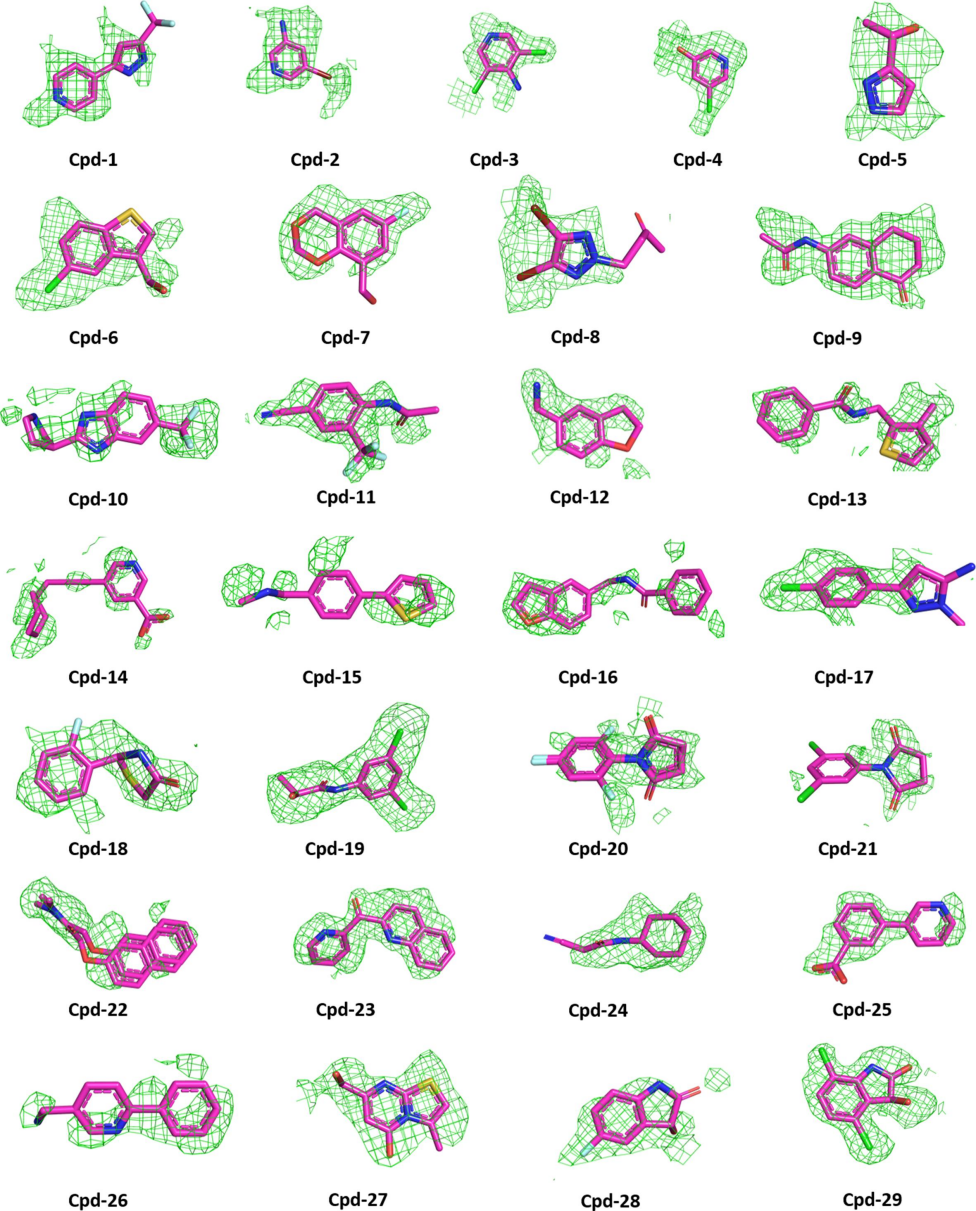
Unbiased omit *F*_o_ − *F*_c_ electron-density maps, contoured at 2σ, are shown in green. Density within 2.4 Å of the ligand molecule is shown. Stick models show C (magenta), N (blue), O (orange red), Br (dark red), S (yellow), Cl (green) and F (light blue) atoms. Cpd-18 was used as a racemic mixture.

**Table 1:** IC_50_ values of cpd-28 and cpd-29 after 30 and 60 min pre-incubation (five concentration points, each measured in duplicate)

Inhibitor	3CL^pro^ IC_50_ (µ*M*)	
	30 min pre-incubation	60 min pre-incubation
Cpd-28	214	228
Cpd-29	109	100
