# Supplementary material for: Fragment-based screening targeting an open form of the SARS-CoV-2 main protease binding pocket
Source: Acta Crystallogr D Struct Biol. 2024 Jan 30;80(Pt 2):123–36. doi: 10.1107/S2059798324000329 (PMC10836397; doi:10.1107/S2059798324000329)
Supplement: Supplementary file 1 [file d-80-00123-sup1.pdf]

Volume 80 (2024)

Supporting information for article:

Fragment-based screening targeting an open form of the SARS-CoV-2 main protease binding pocket

Chia-Ying Huang, Alexander Metz, Roland Lange, Nadia Artico, Céline Potot, Julien Hazemann, Manon Müller, Marina Dos Santos, Alain Chambovey, Daniel Ritz, Deniz Eris, Solange Meyer, Geoffroy Bourquin, May Sharpe and Aengus Mac Sweeney

S1. Cloning and expression

SARS-CoV-2 $3CL^{pro}$ expression as a Hexa-histidine-SUMO-fusion protein

amino acid sequence:

**MGSSHHHHHHGSGLVPRGSASMSDSEVDQEAKPEVKPEVKPETHINLKVSDGSS
EIFFKIKKTTPLRRLMEAFAKRQGKEMDSLRFLYDGIRIQADQTPEDLDMEDND
IIEAHREQIGGSGFRKMAFPSGKVEGCMVQVTCGTTTLNGLWLDDVVYCPRHV
ICTSEDMLNPNYEDLLIRKSNHNFLVQAGNVQLRVIGHSMQNCVLKLKVDTANP
KTPKYKFVRIQPGQTFSVLACYNGSPSGVYQCAMRPNFTIKGSFLNGSCGSVGF
NIDYDCVSFCYMHHMELPTGVHAGTDLEGNFYGPFVDRQTAQAAGTDTTITVN
VLAWLYAAVINGDRWFLNRFTTTLNDFNLVAMKYNYEPLTQDHVDILGPLSAQ
TGIAVLDMCASLKELLQNGMNGRTILGSALLEDEFTPFDVVRQCSGVTFQ**

Black: Hexa-His tag and linker ; Blue: SUMO ; Red: $3CL^{pro}$

S2. Hexahistidine SUMO-$3CL^{pro}$

425 aas; Mol Wt 47194.9, Isoelectric Pt (pI) 5.80

Amino acid sequence “Backtranseq”; *E. coli* codon usage high

https://www.ebi.ac.uk/Tools/st/emboss_backtranseq/

S3. DNA synthesized (Genscript) and cloned into pET29a+

CATATGGGTTCTTCTCACCACCACCACCACCACGGTTCTGGTCTGGTTCCGCGTG
GTTCTGCGTCTATGTCTGACTCTGAAGTTGACCAGGAAGCGAAACCGGAAGTTAA
ACCGGAAGTTAAACCGGAAACCCACATCAACCTGAAAGTTTCTGACGGTTCTTCT
GAAATCTTCTTCAAAATCAAAAAAACCACCCCGCTGCGTCGTCTGATGGAAGCGT
TCGCGAAACGTCAGGGTAAAGAAATGGACTCTCTGCGTTTCCTGTACGACGGTAT
CCGTATCCAGGCGGACCAGACCCCGGAAGACCTGGACATGGAAGACAACGACAT
CATCGAAGCGCACCGTGAACAGATCGGTGGTTCTGGTTTCCGTAAAATGGCGTTC
CCGTCTGGTAAAGTTGAAGGTTGCATGGTTCAGGTTACCTGCGGTACCACCACCC

TGAACGGTCTGTGGCTGGACGACGTTGTTTACTGCCCGCGTCACGTTATCTGCAC
CTCTGAAGACATGCTGAACCCGAACTACGAAGACCTGCTGATCCGTAAATCTAAC
CACAACTTCCTGGTTCAGGCGGGTAACGTTCAGCTGCGTGTTATCGGTCACTCTA
TGCAGAACTGCGTTCTGAAACTGAAAGTTGACACCGCGAACCCGAAAACCCCGA
AATACAAATTCGTTCGTATCCAGCCGGGTCAGACCTTCTCTGTTCTGGCGTGCTA
CAACGGTTCTCCGTCTGGTGTTTACCAGTGCGCGATGCGTCCGAACTTCACCATC
AAAGGTTCTTTCCTGAACGGTTCTTGCGGTTCTGTTGGTTTCAACATCGACTACGA
CTGCGTTTCTTTCTGCTACATGCACCACATGGAACTGCCGACCGGTGTTCACGCG
GGTACCGACCTGGAAGGTAACTTCTACGGTCCGTTCGTTGACCGTCAGACCGCGC
AGGCGGCGGGTACCGACACCACCATCACCGTTAACGTTCTGGCGTGGCTGTACGC
GGCGGTTATCAACGGTGACCGTTGGTTCCTGAACCGTTTCACCACCACCCTGAAC
GACTTCAACCTGGTTGCGATGAAATACAACTACGAACCGCTGACCCAGGACCAC
GTTGACATCCTGGGTCCGCTGTCTGCGCAGACCGGTATCGCGGTTCTGGACATGT
GCGCGTCTCTGAAAGAACTGCTGCAGAACGGTATGAACGGTCGTACCATCCTGG
GTTCTGCGCTGCTGGAAGACGAATTCACCCCGTTCGACGTTGTTCGTCAGTGCTC
TGGTGTTACCTTCCAGTAATAG**GGATCC**

S4. Storage buffer (X-ray)

20 mM Tris-HCl pH 7.8, 150 mM NaCl, 1 mM TCEP, 1 mM EDTA

S5. Storage buffer (Rapidfire MS)

20 mM Tris-HCl pH 7.8, 150 mM NaCl, 1 mM TCEP, 1 mM EDTA, 5% glycerol

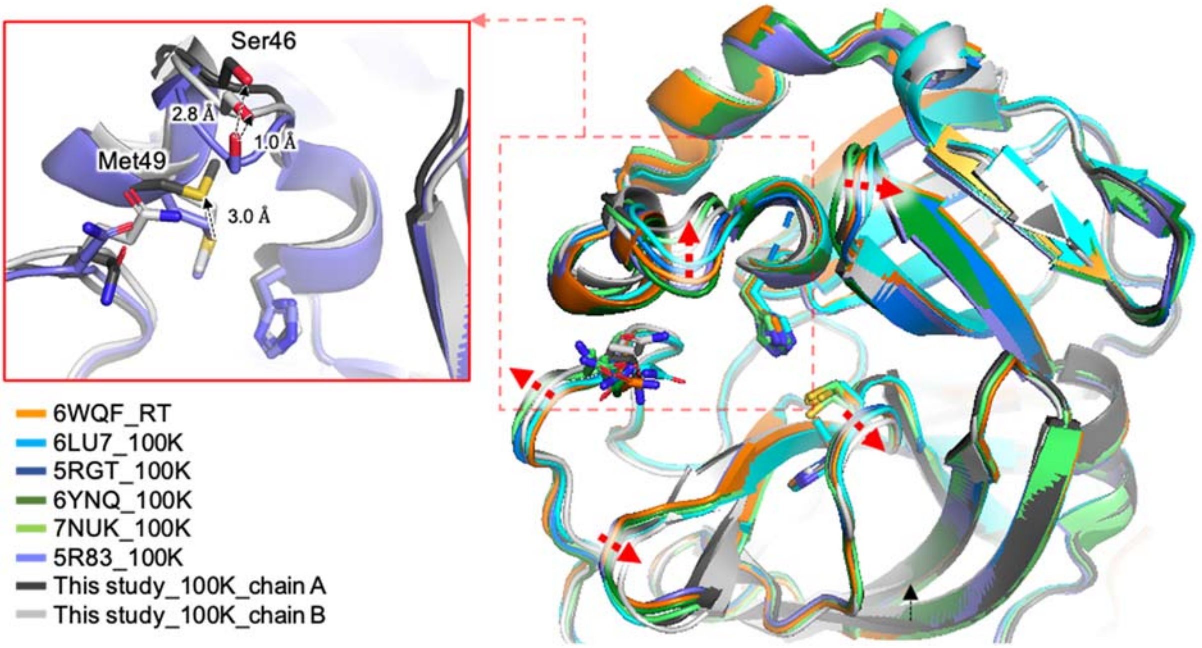

Figure S1 Comparison of the active site cavity conformations of different 3CL^{pro} structures. The protein structures are shown in cartoon representation, with selected active site residues shown in a stick representation. The distance is indicated by dashed arrows and corresponding residues are highlighted.

6WQF: Kneller et al 2020 (https://doi.org/10.1038/s41467-020-16954-7)

6LU7: Jin et al 2020 (https://doi.org/10.1038/s41586-020-2223-y)

5RGT: Zaidman et al 2021 (https://doi.org/10.1016/j.chembiol.2021.05.018)

6YNQ: Gunther et al., 2021 (https://doi.org/10.1126/science.abf7945)

7NUK: Sutanto et al., 2021 (https://doi.org/10.1002/anie.202105584)

5R83: Douangamath et al., 2020 (https://doi.org/10.1038/s41467-020-18709-w)

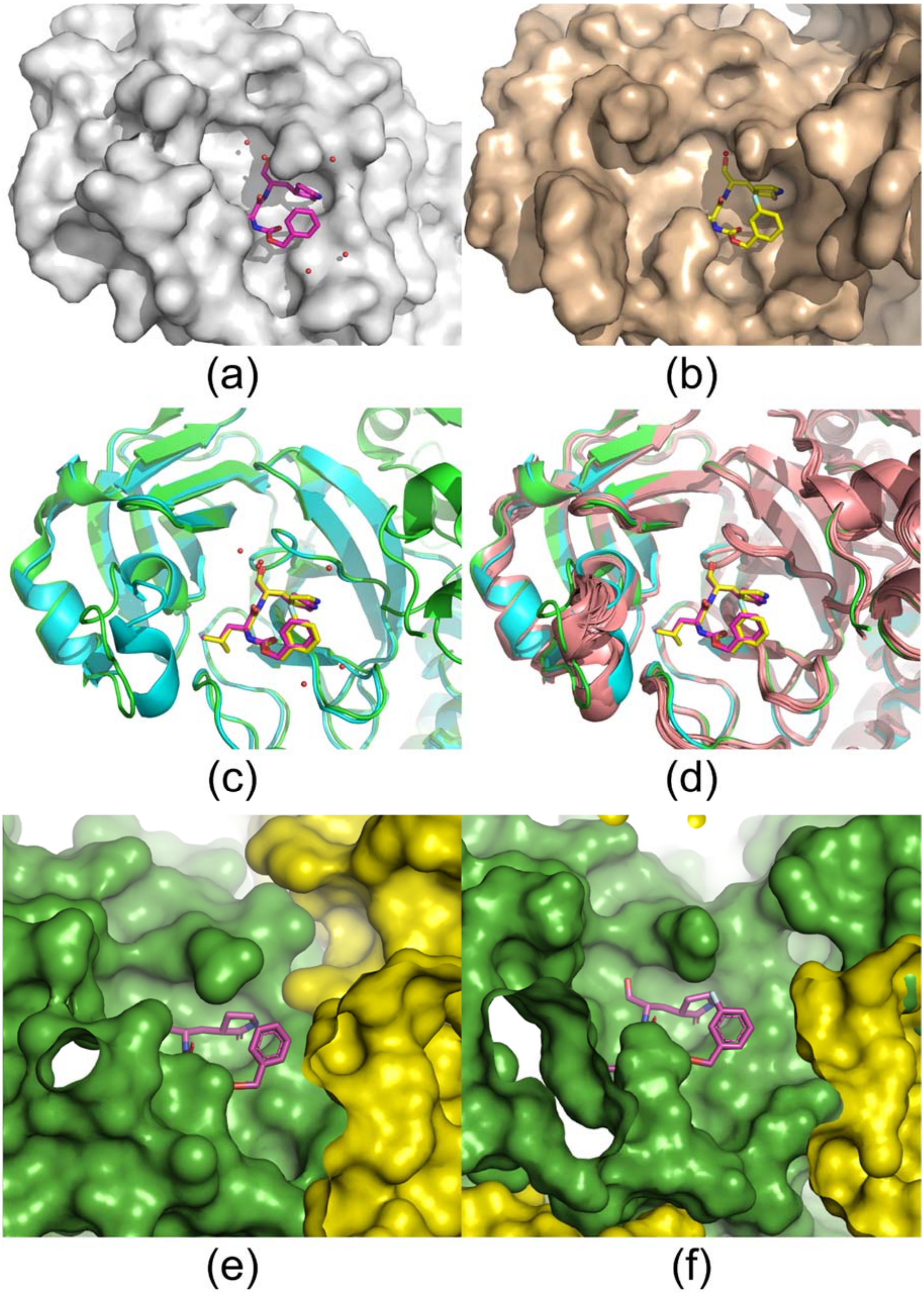
(a)
(b)
(c)
(d)
(e)
(f)

Figure S2 Pocket openness and accessibility. Depicted are (a) the pocket conformations of PDB codes 7c6u ("Type 1" crystals) and (b) 7lcr ("Type 2" crystals) with the protein in surface representation and the ligands as stick models. In (c) both structures are superimposed as cartoon models highlighting the similarly open pocket conformation, except for the left side rim (Cys44 to Ans51, see arrow) that is disordered in 7lcr (green) compared to the helix found for this stretch in 7c6u (cyan). In (d), the additional superimposition with all fragment-bound structures reported in the study (rose cartoon) reveals similar pocket conformations with the majority exhibiting an ordered rim section. Also the crystal packing close to the pocket, and hence the accessibility, is similar for both crystal forms as shown for (e) 7c6u and for (f) 7lcr, with surface representations for the asymmetric unit containing the pocket of interest (green) and close-by symmetry mates (yellow).

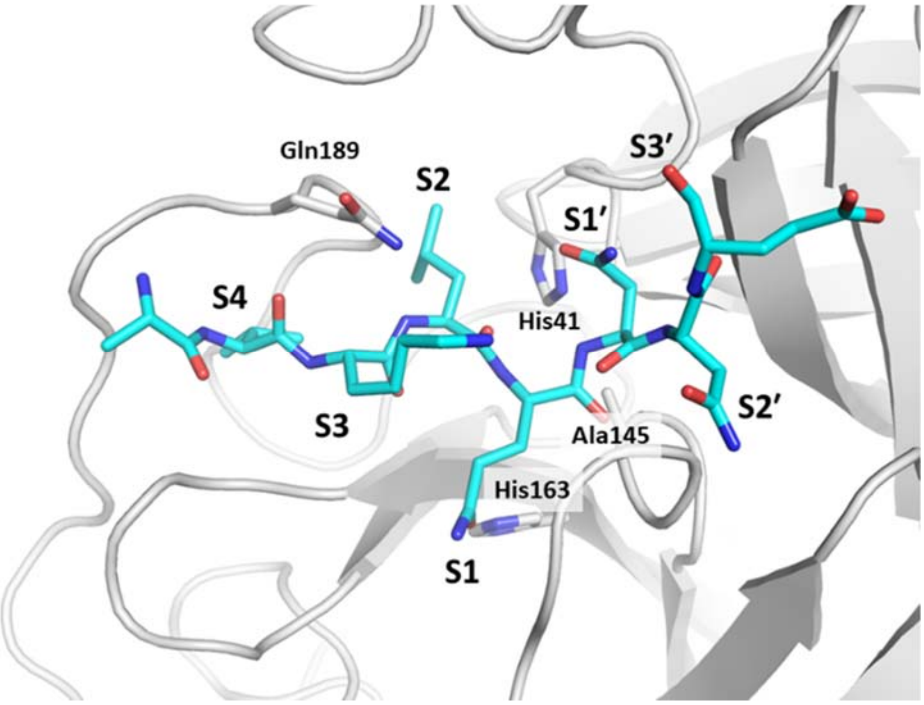

Figure S3 A substrate peptide bound to a catalytically inactive mutant of 3CL^{pro} (PDB entry 7mgr). The protein is shown in cartoon representation in grey, and the substrate peptide is shown in stick representations in blue. Stick models show C (cyan), N (blue) and O (red) atoms.

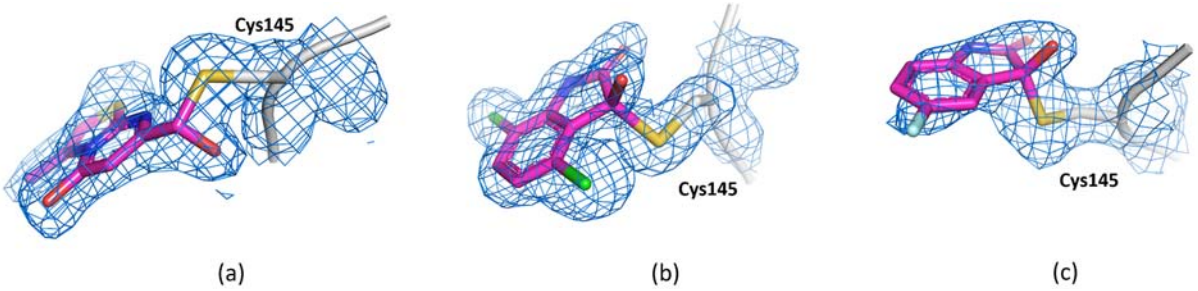

Figure S4 2Fo-Fc electron density contoured at one sigma, showing the covalent bond of (a) cpd-27 (b) cpd-28 and (c) cpd-29 to Cys145 of 3CL^{pro}. The protein is shown in cartoon representation in grey, with the Cys145 side chain in stick representation, and the inhibitors are shown in stick representations in magenta. Stick models show C (magenta), N (blue), O (red), F (light blue) and Cl (green) atoms.

Table S1. Data collection and refinement statistics.

	cpd-1	cpd-2	cpd-3	cpd-4	cpd-5	cpd-6	cpd-7	cpd-8	cpd-9	cpd-10	cpd-11	cpd-12	cpd-13	cpd-14	cpd-15
**Data collection***															
PDB code	7GRE	7GRF	7GRG	7GRH	7GRI	7GRJ	7GRK	7GRL	7GRM	7GRN	7GRO	7GRP	7GRQ	7GRR	7GRS
Resolution range (Å)	56.82-1.66 (1.69-1.66)**	56.68-1.84 (1.88-1.84)	56.81-1.54 (1.55-1.54)	56.94-1.87 (1.91-1.87)	57.09-1.79 (1.82-1.79)	46.36-1.74 (1.77-1.74)	56.25-1.80 (1.83-1.80)	56.77-1.68 (1.7-1.68)	56.65-1.70 (1.73-1.70)	56.46-1.92 (1.97-1.92)	56.68-1.55 (1.57-1.55)	56.06-1.56 (1.58-1.56)	56.70-1.67 (1.70-1.67)	52.04-1.68 (1.71-1.68)	45.93-1.47 (1.49-1.47)
Space group	$P2_12_12_1$	$P2_12_12_1$	$P2_12_12_1$	$P2_12_12_1$	$P2_12_12_1$	$P2_12_12_1$	$P2_12_12_1$	$P2_12_12_1$	$P2_12_12_1$	$P2_12_12_1$	$P2_12_12_1$	$P2_12_12_1$	$P2_12_12_1$	$P2_12_12_1$	$P2_12_12_1$
Cell dimensions a, b, c (Å)	67.79 99.93 104.20	67.71 99.47 103.64	67.78 100.18 104.17	67.97 101.02 104.27	68.1 101.23 104.7	68.03 100.96 104.38	67.83 100.65 104.01	67.78 99.57 103.9	67.61 99.63 103.81	67.31 99.63 103.69	67.62 99.49 103.93	67.75 99.82 103.89	67.73 99.35 103.68	67.77 100.55 104.08	67.64 99.77 103.48
Total reflections	850712 (39169)	581538 (26315)	1169474 (57088)	575937 (29190)	712847 (36987)	672153 (32279)	712405 (35462)	936775 (40174)	902494 (44166)	536788 (30350)	958735 (43164)	929045 (45450)	801741 (36103)	799019 (37171)	1128370 (54773)
Unique reflections	62374 (3120)	42769 (2139)	85975 (4300)	42625 (2131)	51558 (2579)	51113 (2557)	52022 (2601)	68538 (3428)	66556 (3329)	41138 (2057)	70623 (3531)	67649 (3382)	60381 (3020)	58288 (2914)	83806 (4191)
Mean I / σ (I)	9.9 (1.7)	8.8 (1.7)	14.9 (1.4)	8.3 (1.7)	11.1 (1.6)	10.7 (1.5)	10.6 (1.6)	11.9 (1.6)	14.2 (1.5)	6.6 (1.5)	11.6 (1.7)	13.7 (1.8)	7.4 (1.7)	13.1 (1.8)	15.6 (1.8)
Wilson B-factor	35.08	38.05	31.77	46.42	39.24	43.216	36.57	34.04	24.17	43.107	33.62	33.51	32.15	37.95	28.82
R-meas	0.22 (2.06)	0.29 (2.08)	0.12 (2.13)	0.21 (2.11)	0.22 (2.15)	0.18 (2.46)	0.21 (2.18)	0.18 (2.19)	0.14 (2.07)	0.38 (2.31)	0.19 (2.18)	0.14 (1.78)	0.30 (2.21)	0.15 (1.72)	0.16 (1.70)
CC1/ 2 (%)	99.7 (39.7)	99.6 (52.0)	99.9 (53.8)	99.7 (42.5)	99.7 (51.1)	99.8 (64.4)	99.8 (54.5)	99.8 (48.0)	99.9 (60.7)	99.0 (33.5)	99.8 (48.6)	99.9 (57.0)	99.5 (39.8)	99.9 (66.7)	99.9 (63.8)
Completeness spherical (%)***	74.6 (15.5)	69.4 (12.4)	81.0 (24.1)	71.0 (13.5)	74.9 (15.0)	68.6 (12.2)	78.1 (16.7)	84.5 (26.3)	96.2 (27.2)	76.6 (18.3)	68.9 (14.2)	67.4 (11.3)	74.0 (18.7)	71.2 (13.0)	70.0 (13.8)
Completeness ellipsoidal (%)	91.3 (46.6)	93.8 (62.9)	94.4 (64.8)	92.8 (56.9)	93.8 (59.4)	91.3 (50.9)	94.4 (57.3)	91.8 (43.4)	86.2 (54.6)	92.3 (68.2)	91.9 (48.8)	94.8 (64.3)	90.4 (39.2)	94.6 (64.0)	95.3 (66.9)
Multiplicity	13.6 (12.6)	13.6 (12.3)	13.6 (13.3)	13.5 (13.7)	13.8 (14.3)	13.2 (12.6)	13.7 (13.6)	13.7 (11.7)	13.6 (13.3)	13.0 (14.8)	13.6 (12.2)	13.7 (13.4)	13.3 (12.0)	13.7 (12.8)	13.5 (13.1)
**Refinement**															
Reflections used in refinement	62370	42759	85946	42624	51554	50966	52012	68505	66548	40980	70608	67641	60376	58285	83797
R_{work}/ R_{free}	0.21/ 0.25	0.20/ 0.24	0.20/ 0.24	0.20/ 0.25	0.21/ 0.26	0.22/ 0.27	0.20/ 0.24	0.20/ 0.23	0.20/ 0.23	0.25/ 0.30	0.19/ 0.23	0.19/ 0.23	0.19/ 0.23	0.21/ 0.25	0.19/ 0.22
Number of non-hydrogen atoms	5324	5142	5313	5063	5110	5091	5176	5258	5118	5074	5394	5362	5241	4970	5574
macromolecules	4722	4687	4730	4694	4664	4691	4670	4721	4686	4658	4777	4724	4693	4706	4734
ligands	58	54	36	89	37	49	35	46	66	59	76	45	47	45	129
solvent	544	401	547	280	409	351	471	491	366	357	541	593	501	219	711
RMS(bonds) (Å)	0.008	0.004	0.007	0.006	0.004	0.004	0.008	0.039	0.020	0.009	0.009	0.022	0.010	0.011	0.009
RMS(angles) (°)	0.96	0.67	0.98	0.86	0.61	0.66	1.03	1.22	1.04	1.08	1.08	1.79	1.17	1.17	1.06
Ramachandran favored (%)	97.86	97.51	98.19	97.35	98.16	97.84	97.84	97.68	98.16	97.16	98.52	98.51	98.50	97.82	98.03
Ramachandran allowed (%)	2.14	2.49	1.81	2.65	1.84	2.16	2.16	2.32	1.84	2.84	1.48	1.49	1.50	2.18	1.97
Ramachandran outliers (%)	0.00	0.00	0.00	0.00	0.00	0.00	0.00	0.00	0.00	0.00	0.00	0.00	0.00	0.00	0.00
Clashscore	4.02	4.26	3.72	5.59	2.90	4.49	4.41	3.71	3.19	7.39	3.95	4.02	5.23	5.65	4.88
Average B-factor	22.00	28.20	25.40	35.80	29.04	34.09	27.97	24.44	28.44	30.21	25.70	24.48	20.81	28.15	23.67
macromolecules	21.12	27.72	24.52	35.25	28.55	33.95	27.31	23.55	27.55	30.28	24.56	23.32	19.95	27.94	21.81
ligands	33.19	41.67	34.50	62.79	39.31	42.38	35.02	35.65	57.47	34.54	46.07	40.07	31.04	43.57	44.11
solvent	28.41	32.01	32.43	36.40	33.70	34.73	33.97	31.92	34.58	28.50	32.92	32.52	27.92	29.38	32.30

	cpd-16	cpd-17	cpd-18	cpd-19	cpd-20	cpd-21	cpd-22	cpd-23	cpd-24	cpd-25	cpd-26	cpd-27	cpd-28	cpd-29
**Data collection***														
PDB code	7GRT	7GRU	7GRV	7GRW	7GRX	7GRY	7GRZ	7GR0	7GR1	7GR2	7GR3	7GR4	7GR5	7GR6
Resolution range (Å)	56.07-1.71 (1.75-1.71)	56.71-1.81 (1.84-1.81)	56.82-1.92 (1.96-1.92)	56.94-1.92 (1.96-1.92)	56.08-1.55 (1.57-1.55)	56.64-1.54 (1.56-1.54)	46.32-1.86 (1.89-1.86)	55.81-1.69 (1.71-1.69)	71.51-1.74 (1.78-1.74)	56.77-1.77 (1.80-1.77)	56.21-1.89 (1.94-1.89)	56.85-1.86 (1.93-1.86)	56.94-1.88 (1.95-1.88)	56.16-1.62 (1.68-1.62)
Space group	$P2_12_12_1$	$P2_12_12_1$	$P2_12_12_1$	$P2_12_12_1$	$P2_12_12_1$	$P2_12_12_1$	$P2_12_12_1$	$P2_12_12_1$	$P2_12_12_1$	$P2_12_12_1$	$P2_12_12_1$	$P2_12_12_1$	$P2_12_12_1$	$P2_12_12_1$
Cell dimensions a, b, c (Å)	67.78 99.79 103.54	67.68 99.44 103.91	67.76 100.04 104.28	67.94 100.67 104.39	67.87 99.57 103.83	67.68 99.16 103.50	67.85 100.65 104.34	67.53 99.15 103.22	67.60 99.03 103.36	67.75 99.52 104	67.25 99.17 102.39	67.74 99.96 104.54	67.92 99.96 104.43	67.99 99.62 104.44
Total reflections	705856 (30303)	780617 (40678)	684097 (35913)	5530002 (27680)	1131064 (55998)	1004192 (43267)	837150 (134943)	923113 (44141)	669878 (28509)	821338 (41410)	440297 (20920)	622297 (32253)	556557 (28282)	1066123 (51031)
Unique reflections	52132 (2608)	56547 (2827)	49742 (2487)	41004 (2050)	84285 (4214)	73820 (3690)	116173 (18522)	67607 (3380)	49438 (2472)	59683 (2984)	34351 (1719)	46079 (2304)	40569 (2029)	77255 (3863)
Mean I / σ (I)	7.3 (1.7)	8.8 (1.6)	9.6 (1.5)	7.4 (1.5)	12.4 (1.4)	12.7 (1.7)	11.36 (1.08)	10.9 (1.6)	9.5 (1.6)	9.8 (1.6)	7.9 (1.6)	10.7 (1.4)	8.2 (1.5)	16.8 (1.6)
Wilson B-factor	38.668	35.63	41.27	41.64	30.34	31.23	40.97	34.70	34.16	32.65	35.20	41.74	42.968	31.97
R-meas	0.27 (2.06)	0.31 (2.27)	0.2 (2.36)	0.27 (2.32)	0.17 (2.16)	0.16 (1.83)	0.12 (1.86)	0.17 (2.32)	0.38 (1.96)	0.35 (2.27)	0.38 (2.75)	0.19 (2.16)	0.22 (2.18)	0.11 (1.88)
CC1/ 2 (%)	99.6 (51.8)	99.6 (49.4)	99.7 (46.9)	99.4 (50.3)	99.8 (55.2)	99.9 (61.1)	99.3 (70.2)	99.8 (48.6)	99.0 (60.9)	99.5 (46.4)	98.7 (98.9)	99.8 (51.4)	99.6 (47.8)	99.9 (63.8)
Completeness spherical (%)***	68.5 (12.1)	88.2 (29.2)	90.7 (38.3)	73.8 (17.0)	82.5 (24.2)	70.8 (12.6)	99.3 (98.1)	86.1 (28.7)	69.40 (14.0)	86.7 (26.7)	61.5 (13.8)	76.8 (18.0)	69.7 (13.7)	85.7 (29.5)
Completeness ellipsoidal (%)	91.9 (54.5)	93.8 (46.8)	93.9 (50.6)	93.6 (62.2)	94.0 (58.5)	95.2 (65.0)	-	92.5 (48.3)	93.40 (56.1)	94.2 (48.0)	79.8 (50.7)	93.7 (57.4)	93.3 (70.3)	94.6 (63.5)
Multiplicity	13.5 (11.6)	13.8 (14.4)	13.8 (14.4)	13.5 (13.5)	13.4 (13.3)	13.6 (11.7)	7.2 (7.2)	13.7 (13.1)	13.5 (11.5)	13.8 (13.9)	12.8 (12.2)	13.5 (14.0)	13.7 (13.9)	13.8 (13.2)
**Refinement**														
Reflections used in refinement	52109	56541	49735	40997	84280	73816	58522	67558	49437	59678	34289	46076	40565	77251
R_{work}/ R_{free}	0.22/ 0.26	0.19/ 0.24	0.19/ 0.24	0.19/ 0.24	0.19/ 0.22	0.18/ 0.22	0.21/ 0.25	0.19/ 0.22	0.20/ 0.25	0.19/ 0.24	0.23/ 0.28	0.23/ 0.28	0.20/ 0.25	0.19/ 0.23
Number of non-hydrogen atoms	5006	5242	5132	5101	5387	5336	5112	5208	5193	5276	5088	5122	4945	5275
macromolecules	4682	4718	4697	4654	4739	4749	4663	4732	4691	4705	4709	4684	4676	4707
ligands	56	52	57	70	82	54	64	41	54	52	35	36	83	69
solvent	268	472	378	377	566	533	385	435	448	519	344	402	186	499
RMS(bonds) (Å)	0.019	0.097	0.012	0.005	0.008	0.015	0.005	0.011	0.066	0.010	0.003	0.004	0.010	0.013
RMS(angles) (°)	0.89	3.37	1.29	0.79	0.97	1.43	0.77	1.22	2.71	1.09	0.52	1.02	1.10	1.40
Ramachandran favored (%)	98.00	98.51	97.84	98.16	97.84	98.01	97.83	98.68	98.84	98.02	96.86	98.17	96.01	98.17
Ramachandran allowed (%)	2.00	1.49	2.16	1.84	2.16	1.99	2.17	1.32	1.16	1.98	3.14	1.83	3.99	1.83
Ramachandran outliers (%)	0.00	0.00	0.00	0.00	0.00	0.00	0.00	0.00	0.00	0.00	0.00	0.00	0.00	0.00
Clashscore	4.06	5.82	6.49	4.93	2.83	3.16	3.33	4.13	3.30	5.00	4.80	6.11	6.71	4.78
Average B-factor	27.15	24.89	32.21	36.26	25.97	24.44	38.24	28.17	25.52	21.72	19.49	33.75	38.80	28.18
macromolecules	26.63	24.02	31.70	35.64	24.62	24.17	37.70	27.33	24.80	20.78	19.42	33.42	38.50	27.02
ligands	49.37	40.19	43.58	51.36	40.14	45.30	46.57	33.39	38.21	32.81	27.24	44.27	59.70	49.64
solvent	31.45	31.84	36.80	41.05	35.22	34.79	43.30	36.82	31.52	29.19	19.72	36.66	36.93	36.18

*Data processing statistics are reported with Friedel pairs separated.

**Statistics for the highest-resolution shell are shown in parentheses.

***With the exception of the cpd-22, which was processed by XDS, datasets were processed and elliptically truncated using the autoPROC STARANISO option.

Table S1 SMILES codes, occupancy and 2mFo-DFc real space correlation coefficient (RSCC, from PDB validation report) of 3CL^{pro} fragment hits. Where multiple copies of the ligand are present in the structure, the highest RSCC value is shown.

* Fragments bound to the protein in different locations.

** Fragments bound to the protein in different conformations at the same location.

Compound	SMILES code	Occupancy	RSCC
**1**	FC(c1cc(-c2ccncc2)n[nH]1)(F)F	0.83	0.76
**2**	Nc1cc(Br)cnc1	0.70/0.61*	0.68
**3**	Nc(c(Cl)cnc1)c1Cl	0.77/0.67*	0.76
**4**	Oc1cc(Cl)cnc1	0.81/0.73*	0.88
**5**	CC(c1ccn[nH]1)=O.Cl	0.84/0.71*	0.89
**6**	OCc1c[s]c(cc2)c1cc2Cl	0.72/0.82*	0.92
**7**	OCc1cc(F)cc2c1OCOC2	0.80	0.88
**8**	CC(CC[n](nc1Br)nc1Br)=O	0.60	0.96
**9**	CC(Nc(cc1)cc(CCC2)c1C2=O)=O	1.00	0.67
**10**	FC(c(cc1)cc2c1[nH]c([C@@H]1CNCC1)n2)(F)F	0.79	0.79
**11**	CC(Nc(cc1)c(C(F)(F)F)cc1C#N)=O	0.74	0.81
**12**	NCc(cc1)cc2c1OCC2	0.79	0.81
**13**	Cc1c(CNC(c2ccccc2)=O)[s]cc1	0.76	0.85
**14**	OC(c1cncc(C#CCC2CCCCC2)c1)=O	1.00	0.82
**15**	CNCc(cc1)ccc1-c1ccc[s]1	0.80/0.86*	0.95
**16**	O=C(c1ccccc1)NCc(cc1)cc2c1OCC2	1.00	0.79
**17**	C[n](c(N)c1)nc1-c(cc1)ccc1Cl	0.76	0.73
**18**	O=C1NC(c(cccc2)c2F)SC1	0.76/0.78*	0.85
**19**	CC(C(Nc1cc(Cl)cc(Cl)c1)=O)O	0.73/0.81*	0.86
**20**	O=C(CCC1=O)N1c(ccc(F)c1)c1F	0.46/0.54**	0.87
**21**	O=C(CCC1=O)N1c1cc(Cl)cc(Cl)c1	1.00	0.84
**22**	CN(C)C(COc1cc2ccccc2cc1)=O	0.80/0.20**	0.79
**23**	O=C(c1ncccc1)c1nc2ccccc2cc1	0.83	0.88
**24**	N#CCC(NC1CCCCC1)=O	0.83	0.86
**25**	OC(c1cc(-c2cnccc2)ccc1)=O	0.73	0.84
**26**	NCc(cc1)cnc1-c1ccccc1	0.85	0.82
**27**	CC(N12)=CSC1=NC(C=O)=CC2=O	0.78	0.85
**28**	O=C(c(cc(cc1)F)c1N1)C1=O	0.84/0.94/0.81*	0.92
**29**	O=C(c(c(N1)c(cc2)Cl)c2Cl)C1=O	1.00/1.00/1.00*	0.88
